# Oxidative Stress-Responsive Apoptosis Inducing Protein (ORAIP) Plays a Critical Role in Dextran Sulfate Sodium-Induced Murine Model of Ulcerative Colitis

**DOI:** 10.3390/medicina60040539

**Published:** 2024-03-26

**Authors:** Akihito Nakajima, Tomoyoshi Shibuya, Takako Yao, Tsutomu Fujimura, Kimie Murayama, Ko Okumura, Akihito Nagahara, Yoshinori Seko

**Affiliations:** 1 Department of Gastroenterology, Graduate School of Medicine, Juntendo University, Bunkyo-ku, Tokyo 113-8421, Japan; anakaji@juntendo.ac.jp (A.N.); nagahara@juntendo.ac.jp (A.N.); 2 Division of Cardiovascular Medicine, Institute for Adult Diseases, Asahi Life Foundation, Tokyo 103-0002, Japan; apr16_taco@yahoo.co.jp; 3 Laboratory of Bioanalytical Chemistry, Tohoku Medical and Pharmaceutical University, Sendai 981-8558, Japan; tfujitsu@tohoku-mpu.ac.jp; 4 Division of Proteomics and Biomolecular Science, BioMedical Research Center, Juntendo University, Bunkyo-ku, Tokyo 113-8421, Japan; murayama@juntendo.ac.jp; 5 Department of Biofunctional Microbiota, Graduate School of Medicine, Juntendo University, Bunkyo-ku, Tokyo 113-8421, Japan; kokumura@juntendo.ac.jp (K.O.); yseko@juntendo.ac.jp (Y.S.)

**Keywords:** ulcerative colitis (UC), Oxidative Stress-Responsive Apoptosis-Inducing Protein (ORAIP), apoptosis, dextran sulfate sodium (DSS), eukaryotic translation initiation factor 5A (eIF5A)

## Abstract

Oxidative stress is implicated in the pathogenesis of various acute disorders including ischemia/reperfusion injury, ultraviolet/radiation burn, as well as chronic disorders such as dyslipidemia, atherosclerosis, diabetes mellitus, chronic renal disease, and inflammatory bowel disease (IBD). However, the precise mechanism involved remains to be clarified. We formerly identified a novel apoptosis-inducing humoral protein, in a hypoxia/reoxygenation-conditioned medium of cardiac myocytes, which proved to be 69th tyrosine-sulfated eukaryotic translation initiation factor 5A (eIF5A). We named this novel tyrosine-sulfated secreted form of eIF5A Oxidative Stress-Responsive Apoptosis-Inducing Protein (ORAIP). To investigate the role of ORAIP in a dextran sulfate sodium (DSS)-induced murine model of ulcerative colitis (UC), we analyzed the effects of in vivo treatment with anti-ORAIP neutralizing monoclonal antibody (mAb) on the DSS-induced disease exacerbation. The body weight in anti-ORAIP mAb-treated group was significantly heavier than that in a mouse IgG-treated control group on day 8 of DSS-treatment ((85.21 ± 1.03%) vs. (77.38 ± 2.07%); (mean ± SE0, *n* = 5 each, *p* < 0.01, *t*-test). In vivo anti-ORAIP mAb-treatment also significantly suppressed the shortening of colon length as well as Disease Activity Index (DAI) score ((5.00 ± 0.44) vs. (8.20 ± 0.37); (mean ± SE), *n* = 5 each, *p* < 0.001, *t*-test) by suppressing inflammation of the rectal tissue and apoptosis of intestinal mucosal cells. These data reveal the pivotal role of ORAIP in DSS-induced oxidative stress involved in an animal model of UC.

## 1. Introduction

Inflammatory bowel disease (IBD) is a chronic inflammatory disorder that affects the gastrointestinal tract. It mainly includes two conditions, Crohn’s disease (CD) and ulcerative colitis (UC). They have distinct differences as well as some similarities. Especially, UC is characterized by inflammation and ulcers in the innermost lining of the colon and rectum. The number of patients with IBD has been significantly increasing in recent years. There were 6.8 million cases of IBD globally [1]. UC is estimated to affect approximately 900,000 people in the United States [2] and more than 220,000 people in Japan [3]. The most common age of onset for IBD is during young adulthood, and since it often coincides with major life events, it can have lasting effects throughout one’s life. IBD has been proposed to be a kind of autoimmune condition; the exact cause is still unknown. In recent years, the development of new drugs such as JAK inhibitors [4,5,6,7], anti-integrin mAb [8,9], anti-IL-12/23 p40 mAb [10,11], anti-IL-23 p19 mAb [12,13], and others has been increasing, in addition to anti-tumor necrosis factor (TNF)-α mAb for the treatment of IBD; however, there are some cases unresponsive to treatment with immunosuppressants and anti-cytokine therapy. This gives overriding priority to the development of novel therapeutic agents targeting another mechanism mediated by distinct pathways. Although evidence has accumulated that oxidative stress and apoptosis are involved in the pathogenesis of cytotoxicity in UC, the precise mechanism had been elusive [14,15,16]. Inflammation in colonic mucosal tissue generates reactive oxygen species/reactive nitrogen species (ROS/RNS), causing mucosal damage and increased permeability of the mucosal barrier [17]. Inflammatory cells, such as neutrophils and macrophages, produce large amounts of ROS. The excessive production of ROS/RNS leads to lipid and protein modifications, DNA damage, apoptosis, and carcinogenesis [17]. There is an imbalance between the generation of ROS/RNS and the antioxidants in UC patients. The endogenous antioxidant system mainly consists of intracellular enzymatic antioxidants, such as superoxide dismutases (SODs), glutathione peroxidase (GPX), and catalase (CAT) [17]. Because antioxidants are key to reducing prooxidants and treating UC, new therapies targeting antioxidants are being developed. For example, it has been reported that oral angiotensin II type 1 blockers and telmisartan administration inhibit ROS production by suppressing NF-κB, cyclooxygenase (COX) 2, and inducible nitric oxide synthase (iNOS) expression in a rat model of colitis [18]. N-acetylcysteine (NAC) contributes to the recovery of UC patients and mouse models. Oral treatments with NAC leads to decreased lipid and protein oxidation, increased glutathione (GSH) and CAT activities, and improved antioxidant status in the colon [19,20]. Additionally, previous studies have shown that short-chain fatty acids (SCFAs) such as acetate, propionate and butyrate, produced by colonic bacteria, play an essential role for antioxidant defense [21]. SCFAs are produced by dietary fibers in the intestine, and dietary fibers intake affects the gut microbiota and the mucosal immune system. Many recent studies suggest that the gut microbiota are associated with UC. Butyrate treatment in UC patients resulted in significantly higher GSH levels and improved mucosal damage [22]. Furthermore, previous studies have demonstrated that mice fed a soluble high-fiber diet had significantly reduced DSS-induced colitis compared to mice fed a no-fiber diet. SCFAs have many functions to prevent inflammatory response in the gut, including antioxidant activity, along with their ability to produce regulator T cells and IgA in the gut. Thus, until recent years, it was believed that reactive oxygen species (ROS) were the primary culprits behind oxidative stress-induced cell damage [23]. However, despite extensive large-scale clinical trials on antioxidants, including free radical scavengers and vitamins, there has been no significant improvement in the outcomes of cardiovascular and cerebrovascular diseases. Similarly, the efficacy of vitamin D therapy for patients with ulcerative colitis remains uncertain [24]. This suggests the existence of unidentified factors other than ROS that contribute to oxidative stress-induced cell injury. In our previous research, we identified a new humoral factor capable of inducing apoptosis in cardiac myocytes subjected to hypoxia/reoxygenation. This factor is a highly hypusinated and tyrosine-sulfated form of eukaryotic translation initiation factor 5A (eIF5A), specifically secreted in response to oxidative stresses such as ischemia/reperfusion, hypoxia/reoxygenation, ultraviolet irradiation, ionizing radiation, heat shock, and blood acidification [25,26]. We have named this post-translationally modified secreted form of eIF5A Oxidative Stress-Responsive Apoptosis-Inducing Protein (ORAIP). Several studies have shown that ORAIP promotes apoptosis. Treatment with an anti-ORAIP mAb significantly reduced ischemia/reperfusion injury in rats [25,26]. Moreover, plasma levels of ORAIP are elevated in patients with chronic kidney disease, atrial fibrillation, chronic heart failure, and hypercholesterolemia [27]. We have also found significantly higher concentrations of ORAIP in the vitreous body of patients with proliferative diabetic retinopathy compared to those with non-proliferative diabetic retinopathy, suggesting a role for ORAIP in oxidative stress-induced retinal injury [28]. These findings indicate that ORAIP could serve as a sensitive diagnostic marker and a promising therapeutic target for oxidative stress-induced cytotoxicity in various disorders.

To investigate the critical role of ORAIP in the mechanism of intestinal epithelial cell injury involved in UC, we analyzed the effects of anti-ORAIP neutralizing mAb on apoptosis in intestinal epithelial cells in a dextran sulfate sodium (DSS)-induced murine model of UC in vivo [29].

## 2. Materials and Methods

### 2.1. Animals

This study was carried out in accordance with the Guide of The Japanese Association of Laboratory Animal Facilities of National University Corporations and with approval of the institutional animal care committee. Ten-week-old C57BL/6J male mice were purchased from Japan SLC (Shizuoka, Japan). Mice were housed under a specific pathogen-free condition at the animal facility of Juntendo University (Tokyo, Japan) and fed a normal diet (CRF-1; Charles River, Wilmington, MA, USA). All animal experiments were approved by the Animal Experimentation Committee of Juntendo University (No. 2022113).

### 2.2. Dextran Sulfate Sodium (DSS)-Induced Colitis

**Figure 1:**
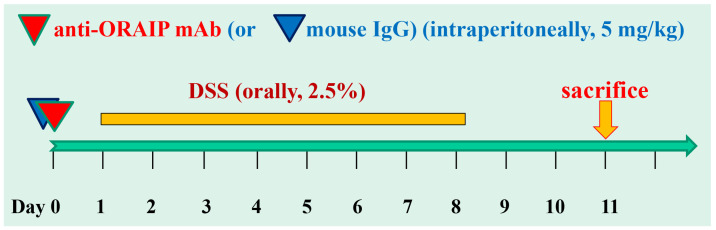
In vivo study design and schedule for DSS-administration and intraperitoneal injection of anti-ORAIP mAb and control mouse IgG.

Mice were randomly assigned to control mouse IgG and anti-ORAIP mAb groups. We determined the necessary and sufficient dosage of 5 mg/Kg for investigating the effectiveness of this mAb. Specifically, based on the former research, dosages of 1, 3, 5, and 10 mg/Kg were tested (*n* = 12 for each group) [25]. In the present study, to minimize animal sacrifices, we conducted the research with a single-dose administration. We injected 5 mg/Kg of anti-ORAIP mAb (*n* = 5) or control mouse IgG (*n* = 5) intraperitoneally on day 0. Then, 2.5% DSS (MW 36000-50000, MP Biomedicals, Santa Ana, CA, USA) dissolved in sterile, distilled drinking water was orally administered from day 1 to day 7. Then, 2.5% DSS was switched to regular drinking water on day 8. The mice were anesthetized by 5% isoflurane inhalation to be euthanized on day 11 (Figure 1). The body weight and colon length of each mouse were measured.

### 2.3. Anti-ORAIP mAb

We formerly reported that the apoptosis-inducing secreted form of eIF5A (known as ORAIP) undergoes two post-translational modifications; hypusination at Lys50 and sulfation at Tyr69. Since the apoptosis-inducing activity of the non-hypusinated secreted form of eIF5A was much lower than that of the hypusinated secreted form [15], we hypothesize that tyrosine-sulfation is essential for receptor binding and signaling as a proapoptotic ligand, while hypusination plays an important role in this process. Therefore, we have developed a neutralizing anti-ORAIP mAb targeting amino acid residues, including these sites. A mouse anti-eIF5A mAb (clone YSP5-45-36) was generated against human eIF5A peptides (amino acid residues 44 to 72, which includes the hypusination site and 69th tyrosine sulfation site, coupled to keyhole limpet hemocyanin (KLH)) as described previously [25]. This mAb reacts with the secreted form of eIF5A (ORAIP).

### 2.4. Immunofluorescence

The immunofluorescence staining of ORAIP was performed using the Tyramide Signal Amplification (TSA) technology for fluorescence (TSA^TM^ Plus Fluorescein Kit, PerkinElmer, Shelton, CT, USA, according to the manufacturer’s instructions). We did double immunostaining for ORAIP and for CD4, CD8, or CD14 in the colon tissue from DSS loading with the mouse IgG-treatment group (DSS-IgG group). The procedures have been described elsewhere [16]. The formalin-fixed paraffin-embedded colon tissue samples were deparaffinized, blocked with 0.3% H_2_O_2_ in methanol for 30 min, and incubated with rabbit anti-CD4 (19068-1-AP), CD8 (17335-1-AP), or CD14 (17000-1-AP) polyclonal antibody (Proteintech Group, Inc., Rosemont, IL, USA) for 1 h at 37 °C. After washing in phosphate-buffered saline (PBS), the sections were incubated with trimethylrhodamine isothiocyanate (TRITC)-labeled anti-rabbit IgG (T6778; Sigma-Aldrich, St. Louis, MO, USA) for 1 h at 37 °C. After washing in PBS, the slides were incubated with horseradish peroxidase (HRP)-labeled anti-ORAIP mAb (YSP5-45-36) for 1 h at 37 °C. After washing in TNT buffer (0.1 mol/L Tris-HCl, pH 7.5, 0.15 mol/L NaCl, 0.05% Tween 20), the sections were blocked with TNB buffer containing a blocking reagent for 30 min. After washing in TNT buffer, the sections were incubated with fluorescein isothiocyanate (FITC)-labeled Tyramide in Amplification Reagent for an appropriate time (3 to 10 min), washed in TNT buffer, then examined and photographed under a fluorescence microscope.

### 2.5. TdT (Terminal Deoxynucleotidyl Transferase)-Mediated dUTP Nick End Labeling (TUNEL) Staining

We used the In Situ Apoptosis Detection Kit (TAKARA BIO Inc., Kusatsu, Japan) followed by diaminobenzidine (DAB) reaction (brown color) for TUNEL staining. Briefly, the frozen colon tissue sections were air-dried and fixed with 4% paraformaldehyde for 30 min at room temperature, followed by inactivation of the endogenous peroxidase using 0.3% H_2_O_2_ in methanol for 30 min at room temperature. After incubation with a permeabilization buffer for 5 min on ice, the sections were subjected to the reaction with TdT enzyme and FITC-conjugated dUTP for 90 min at 37 °C. After washing in PBS, the sections were incubated with HRP-labeled anti-FITC for 30 min at 37 °C. After washing in (0.1% Tween-20 in Tris-buffered saline), the sections were subjected to a DAB reaction for 5 to 10 min at room temperature, and then washed in distilled water (DW). Then, the sections were counterstained with 3% methyl green.

### 2.6. Statistical Analysis

Data were analyzed using *t*-tests to compare between the mouse IgG-treated control group and the anti-ORAIP mAb-treated group. *p* values < 0.05 were considered statistically significant. The results are shown as (mean ± standard error [SE]).

## 3. Results

### 3.1. In Vivo Treatment with Anti-ORAIP mAb Suppressed Body Weight Loss, Colon Shortening, and Disease Activity

To investigate the effects of in vivo treatment with anti-ORAIP mAb on the DSS-induced disease exacerbation, we measured the body weight and colon length.

**Figure 2:**
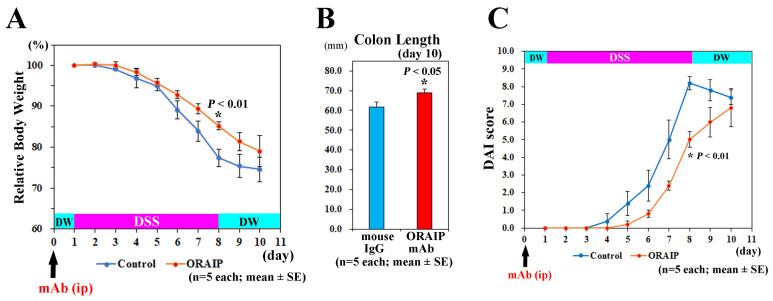
Effects of anti-ORAIP mAb on body weight loss, shortening of colon length, and disease activity. (**A**,**C**) Time course of the relative body weight (**A**) and Disease Activity Index (DAI) score (**C**) in anti-ORAIP mAb-treated group and mouse IgG-treated control group. (**B**) Colon length in the two groups at sacrifice on day 10. The differences between the mouse IgG-treated control group and anti-ORAIP mAb-treated group were significant (* *p* < 0.01 (**A**), * *p* < 0.05 (**B**) and * *p* < 0.01 (**C**), respectively).

As shown in Figure 2A, the body weights of DSS-treated mice in both groups continued to decrease during DSS-treatment until sacrifice at 2 days after the discontinuation of DSS treatment. The decrease in body weight of mice in the anti-ORAIP mAb-treated group was less than in the mouse IgG-treated control group, and the differences in body weight loss between both groups reached a maximum level on day 8 of DSS treatment. The body weight in the anti-ORAIP mAb-treated group was significantly heavier than that in the mouse IgG-treated control group on day 8 of DSS-treatment ((85.21 ± 1.03%) vs. (77.38 ± 2.07%); (mean ± SE), *n* = 5 each, *p* < 0.01, *t*-test). Anti-ORAIP mAb-treatment also significantly suppressed the shortening of colon length. The colon length in anti-ORAIP mAb-treated group was significantly longer than that in mouse IgG-treated control group at sacrifice on day 10 ((69.00 ± 1.92 mm) vs. (61.60 ± 2.54 mm); (mean ± SE), *n* = 5 each, *p* < 0.05, *t*-test) (Figure 2B). In this experimental design, because a recovery period was included after discontinuing DSS administration, it is speculated that the difference would be further pronounced if colon length was measured on day 8. Next, we analyzed the effects of anti-ORAIP mAb-treatment on Disease Activity Index (DAI) score, consisting of weight loss, consistency of stool, and degree of blood in stool, as we previously described [27]. For weight loss: 0—no loss; 1—5–10% loss; 2—10–15% loss; 3—15–20% loss; and 4—20% weight loss. For stool consistency: 0—normal; 1—mild loose stool; 2—moderate loose stool; and 3—diarrhea. For bleeding: 0—no blood; 1—presence of blood; and 2—gross blood. As shown in Figure 2C, the DAI score in the anti-ORAIP mAb-treated group was significantly lower than that in the mouse IgG-treated control group on day 8 of DSS-treatment ((5.00 ± 0.44) vs. (8.20 ± 0.37); (mean ± SE), *n* = 5 each, *p* < 0.001, *t*-test). Due to partial recovery from the peak inflammation on day 8, the differences in the body weight and DAI score between the groups became smaller.

### 3.2. In Vivo Treatment with Anti-ORAIP mAb Suppressed Inflammation of Rectal Tissue and Apoptosis of IELs

**Figure 3:**
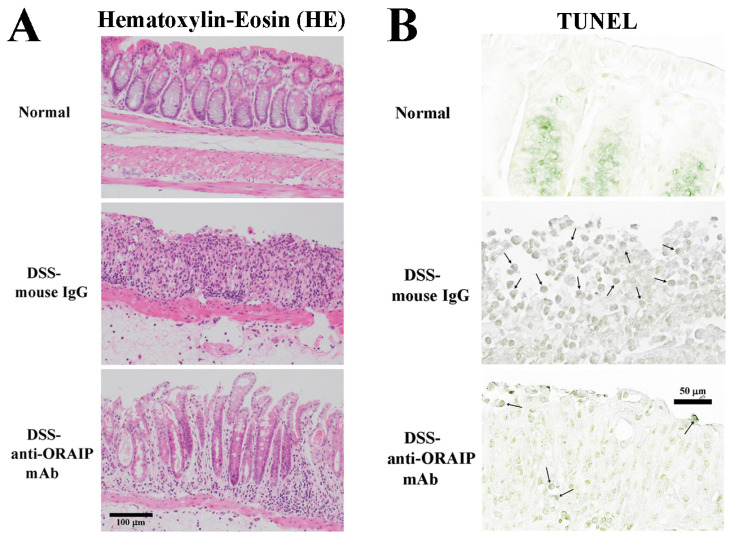
Effects of anti-ORAIP mAb on inflammation and apoptosis in mucosal tissue. (**A**) and (**B**); hematoxylin–eosin (HE) staining (**A**), and TUNEL with methylgreen-nuclear staining ((**B**) arrows indicate apoptotic cells) of intestinal mucosal tissues from normal control group (Normal), DSS-IgG group, and DSS-anti-ORAIP mAb group.

The colon wall tissues of the normal control group (Normal), DSS-IgG group, and DSS loading with anti-ORAIP mAb treatment group (DSS-anti-ORAIP mAb group) were stained with hematoxylin–eosin (HE) or TUNEL (and nuclear staining with methyl green), as shown in Figure 3 (panel A and B, respectively). In HE staining, the normal control group maintained the glandular structure of the villi, and the epithelial cells were aligned. However, in the DSS-IgG group, the glandular structure was absent, and the arrangement of epithelial cells was completely destroyed. In contrast, in the DSS-anti-ORAIP mAb group, both the glandular structure and the arrangement of epithelial cells were partially preserved. Furthermore, in TUNEL staining, most cells in the normal control group were negative, whereas in the DSS-IgG group, numerous epithelial cells and others were positive. Thus, it is evident that the administration of anti-ORAIP mAb apparently suppressed the destruction of mucosal epithelial cells (induction of apoptosis)undergoing apoptosis caused by DSS loading (Figure 3B, middle panel, arrows). In the DSS-anti-ORAIP mAb group, apoptosis was observed only in a very small number of cells (Figure 3B, lower panel, arrows) Thus, it is evident that the administration of anti-ORAIP mAb apparently suppressed the destruction of mucosal epithelial cells (induction of apoptosis) caused by DSS-loading.

**Figure 4:**
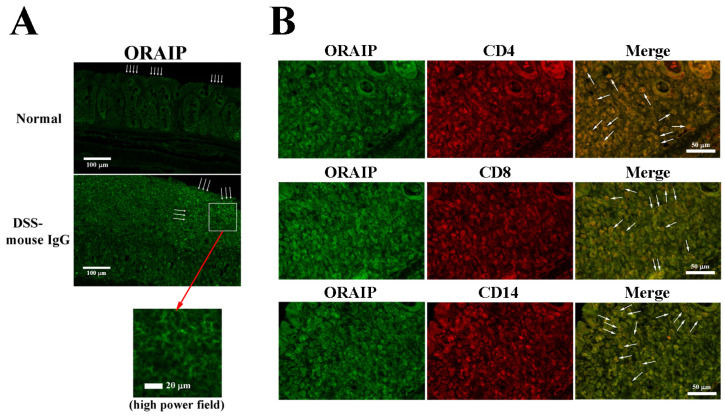
Expression of ORAIP in mucosal tissues with DSS-induced inflammation. (**A**) The immunofluorescence staining of ORAIP expression (arrows) in the intestinal mucosal tissues of the normal control group (Normal) and the DSS-IgG group. (**B**) ORAIP-expressing inflammatory cells in the intestinal mucosal tissue. Double-immunostaining for ORAIP (labeled with FITC) or CD4, CD8, and CD14 (labeled with TRITC) of the intestinal mucosal tissue from a mouse in the DSS-IgG group.

Figure 4A shows the results of the immunofluorescence staining of ORAIP expression in the colon wall tissues of the normal control group (Normal) and the DSS-IgG group. In the epithelial cells on the mucosal surface of the normal control group, a minimal expression of ORAIP was observed (indicated by arrows). In contrast, in the DSS-IgG group, a significant expression of ORAIP was detected in a cluster of cells, presumably densely populated LPLs (middle panel, indicated by arrows; lower panel, high power field). This suggests that oxidative stress was induced in the intestinal mucosal tissues by DSS-loading, leading to colitis with massive infiltration by ORAIP-expressing lamina propria lymphocytes (LPLs), resulting in apoptosis of the mucosal epithelial cells. Double-immunostaining for ORAIP (labeled with FITC) and for CD4, CD8, or CD14 (labeled with TRITC) in the colon wall tissues of the DSS-IgG group revealed a massive infiltration by CD4+ and CD8+ T-cells and CD14+ macrophages strongly expressing ORAIP on their surfaces (Figure 4B). This suggests a possibility that infiltrating immune cells induce apoptosis of the colon wall cells (such as intestinal epithelial cells (IELs)) by secreting ORAIP. In the mucosal tissues from animals of the DSS-anti-ORAIP mAb group, it is thought that the administered anti-ORAIP mAb bound to the expressed ORAIP proteins on the cell surfaces. This would interfere critically with the subsequent immunohistochemical staining using anti-ORAIP mAb, making it impossible to assess the expression of ORAIP impartially. Therefore, we omitted the DSS-anti-ORAIP mAb group from the study.

## 4. Discussion

The present study demonstrates that in vivo treatment with a neutralizing anti-ORAIP mAb clearly reduced the body weight loss, colon shortening, apoptosis in intestinal mucosal cells, and disease activity in a DSS-treated murine model of UC, suggesting that ORAIP rather than ROS plays a pivotal role in DSS-induced intestinal cell injury. The possible mechanisms of action for anti-ORAIP mAb are considered to be the following. First, infiltrating lymphocytes secrete ORAIP, which induces apoptosis in intestinal cells in a paracrine manner. We previously reported that ORAIP-expressing CD4+, CD8+, or CD14+ cells infiltrated the atherosclerotic arterial tissues of patients with heterozygous familial hypercholesterolemia, suggesting a possibility that infiltrating immune cells (such as T-cells and macrophages) induce apoptosis of the arterial smooth muscle cells by secreting ORAIP [27]. In the present study, we have also demonstrated the significant expression of ORAIP on many infiltrating LPLs in vivo, suggesting a critical role of oxidative stress in DSS-induced intestinal mucosal cell injury through the secretion of ORAIP, as in the atherosclerotic arterial tissue. The precise mechanism of oxidative stress generation at the site of inflammation, such as atherosclerotic arterial wall and DSS-loaded intestinal mucosal tissue, remains unknown, and needs to be clarified. Second, we cannot exclude the possibility that intestinal epithelial cells themselves secrete ORAIP in response to oxidative stress, inducing apoptosis in intestinal cells in an autocrine manner. Third, it is speculative, but it is possible that suppressing the ORAIP/ORAIP–receptor pathway could suppress the oxidative stress generation and progression induced by some unknown mechanisms. The precise mechanism responsible for initiating ORAIP secretion through upstream oxidative stress sensing remains uncertain, yet it seems unlikely to be ROS-induced, given the notably quicker timeframe of ORAIP secretion compared to ROS production in the case of ORAIP secretion induced by ischemia/reperfusion [26,30]. This suggests that the ORAIP-mediated signaling pathway is distinct from the ROS-mediated signaling pathway. Therefore, the effects of combination therapy using anti-ORAIP, anti-ROS, and anti-cytokine (such as TNF-α) agents may be additive [31]. Because ORAIP-mediated apoptotic signaling is partially mediated by the JAK/STAT pathway [25], new drugs such as JAK inhibitors may at least partially suppress ORAIP-mediated apoptosis induction. Our previous studies and the present study show that anti-ORAIP mAb critically reduces oxidative stress-induced apoptotic cell injury mediated by physicochemical stimulation, including hypoxia/reoxygenation, high glucose, doxorubicin, and DSS. This suggests that ORAIP-mediated apoptotic cell death may be a common pathway among various types of oxidative stresses and in various types of cells [25,26,32]. It is known that patients with UC have a higher risk of developing colorectal cancer. DNA damage induced by the oxidative stress involved in UC has been thought to be one of the major causes of carcinogenesis, because chronic inflammation generates oxidative stress-induced damage to DNA resulting in the activation of tumor-promoting genes and/or inactivation of tumor suppressor genes [33,34,35]. We are investigating the ORAIP-mediated apoptotic signaling pathway, including cell-surface receptors for ORAIP (that is, ORAIP-receptor; unpublished data) and downstream signaling cascades such as the Jak/STAT pathway, MEK/ERK pathway, and so on. It appears that in response to oxidative stress, ROS-mediated signaling rather than ORAIP-mediated signaling plays major roles in causing DNA damage that leads to carcinogenesis [36,37]. For doxorubicin-induced apoptosis in cardiac myocytes, it seems that both ROS-mediated and ORAIP-mediated apoptotic signaling contribute independently to the total cell injury induced by doxorubicin [32,38]. This is supported by our former findings that the anti-ORAIP mAb treatment was less effective in doxorubicin-induced apoptosis than that in high glucose-induced apoptosis, suggesting a bigger role of ROS in doxorubicin than in high glucose [32]. In the near future, we aim to utilize three-dimensional structural insights into the (ORAIP/ORAIP-receptor) binding site to devise novel therapeutic approaches, including decoy ORAIP-receptor constructs, medium-sized peptides, and small-molecule compounds. These modalities aim to inhibit (ORAIP/ORAIP-receptor) binding to protect against ORAIP-mediated apoptosis induction.

The present study has revealed a novel and critical therapeutic target for the oxidative stress involved in an animal model of UC; however, DSS-induced UC in animals typically develops rapidly after DSS-loading, representing certain histopathological features of human UC, and relatively quickly regresses after the withdrawal of DSS-loading. In contrast, human UC is characterized by the chronic nature of the disease, with long periods of remission and relapse, in which genetic, immunological, and environmental etiology is implicated. Although the present study has revealed the role of ORAIP especially in the aspect of oxidative stress-induced cell injury in DSS-induced colitis, further investigation with multi-dimensional approaches is needed to derive a comprehensive understanding of human UC. For a current therapeutic strategy for UC, 5-aminosalicylic acid (5-ASA) and corticosteroids are initially used to treat mild to moderate UC. If remission is still not achieved, the use of biological agents such as anti-TNF, IL-12/23 inhibitors, α4β7 integrin inhibitors and JAK inhibitors has been considered [39]. According to the OCTAVE trials and the U-ACHIEVE trial, patients receiving tofacitinib and Upadacitinib showed significant clinical remission compared to placebo [4,6]. While JAK inhibitors have shown effectiveness, in the OCTAVE trials [4] and the U-ACHIEVE trial [6], only 40.6% of patients receiving 10 mg of tofacitinib and 52% patients receiving 30 mg of upadacitinib maintained clinical remission at 52 weeks, respectively. The efficacy of these agents is partial and ineffective in the remainder of patients. There may be cases where sufficient effects are not obtained, or even if there are effects, secondary nullification may occur and the effects may disappear. The ORAIP/ORAIP-receptor pathway at least partially includes downstream JAK/STAT signaling pathways [25], and is distinct from the ROS-mediate pathway. Our previous studies have shown that anti-ORAIP mAb inhibits oxidative stress-induced apoptosis more effectively than conventional therapies targeting ROS to mediate major aspects of oxidative stress disorders. Due to its apparently different mechanism from conventional therapies, anti-ORAIP therapy can be used in combination with them, offering the potential for synergistic effects. In particular, in diseases where effective treatments have not been available, it has the potential to be a groundbreaking therapy. Currently, there are no effective therapeutic drugs focusing on anti-oxidative stress, but anti-ORAIP mAb holds potential as a new antibody drug for UC.

## 5. Conclusions

Oxidative stress is implicated in the pathogenesis of UC; however, the precise mechanism is unknown. The present study has revealed the pivotal role of ORAIP in the development of DSS-induced murine colitis, suggesting the critical involvement of oxidative stress-induced cell injury. However, further investigations are needed to develop a comprehensive understanding of human UC and ensure the clinical application of anti-ORAIP therapy in the future.

## 6. Future Directions

We plan to use human samples, such as specimens obtained through colonoscopy biopsy and surgery, as well as blood samples, to develop novel markers for disease activity. Our plan involves analyzing the expression of ORAIP/ORAIP-receptor in affected colon tissues and the plasma levels of ORAIP in patients with IBD. In the next phase, we intend to develop new drug modalities targeting the ORAIP receptor, such as decoy ORAIP receptors, medium-sized peptides, small-molecule compounds, and neutralizing mAb that can protect against ORAIP-mediated apoptosis induction. Since the ORAIP/ORAIP-receptor pathway is distinct from cytokines, immune cells, and ROS-signaling, anti-ORAIP therapy may merit clinical application either compared to or in combination with other conventional therapies for IBD.

## Data Availability

We have no other data supporting the results.
